# Indian endocrine surgery websites are they comparable?

**DOI:** 10.1016/j.amsu.2020.11.021

**Published:** 2020-11-18

**Authors:** Vnssvams Mahalakshmi D, Mayilvaganan Sabaretnam, Dabeer Warsi, P.R.K. Bhargav, Aromal Chekavar, Amit Agarwal

**Affiliations:** a Department of Endocrine Surgery, Sanjay Gandhi Postgraduate Institute of Medical Sciences, Raebareily Road, Lucknow, 226 014, India; b School of Telemedicine, Sanjay Gandhi Postgraduate Institute of Medical Sciences, Rae Bareilly Road, Lucknow, 226 014, India; c Consultant Endocrine Surgeon, India

**Keywords:** Endocrine surgery, Website, Thyroid, Parathyroid

## Abstract

**Introduction:**

In modern day surgical practice, patients and their relatives garner more knowledge through websites rather than direct interaction with the consultant physician. We aimed to assess whether Indian endocrine surgery websites matched with their counterparts abroad.

**Materials & methods:**

We identified 60 endocrine surgery websites worldwide and 12 endocrine surgery websites maintained by trained endocrine surgeons from India. The website parameters, demographic data of the websites, rank, and other parameters were assessed using a professional website (www.Alexa.com). An endocrine surgeon along with a technical website advisor rated the content, presentation, and likes from a scale of 1–5 (1 minimum score and 5 maximum score).

**Results:**

A total of 72 individual endocrine surgery websites, out of which 60 were from abroad and 12 were from India, were analyzed. A majority of foreign websites were ranked (43/60), whereas 2/12 Indian websites were ranked (P < 0.0001). Foreign websites had a better landscape profile. Except for pancreatic facts, which were significantly different (P = 0.006) between Indian and foreign websites, there was no significant difference in thyroid facts, parathyroid facts, adrenal facts, photographs, videos, postop advice, contact information, publications, and complications.

**Conclusion:**

Most parameters were comparable in both groups. Postoperative advice and complications were present in only a few websites. A well-designed endocrine surgery website can aid both the patient and the treating physician.

## Highlights

• Websites can help patients garner knowledge.
• Well designed and unbiased website can be a good tool.
• Websites can safe unnecessary discussion with the consultant.
• Website can help take decisions.

## 1 Introduction

With the present day 4G, the 4th generation of mobile technology with an increased date speed that is up to 10 times faster than the standard 3G [1], websites with pertinent information regarding diseases and surgeons have replaced the good old “word of mouth” recommendation of the surgeon [2], and this in turn helps the patient and their relatives garner adequate information regarding the disorder and the intervention the patient needs from the websites. With the advent of telemedicine facilities and as treating physicians get trained in the technological aspect of websites and in other technologies, many innovative animations and creative website designs have started to emerge. With the present day technology aiding the knife happy surgeon, the endocrine surgeon is not only a surgeon scientist but also one who adores the role of a technological resource person for developing an aesthetically and, at the same time, informative website. These websites can provide adequate information and, at the same time, avoid unnecessary discussions with relatives and help save crucial time for the busy consultant. Endocrine surgery itself is special since most of the patients need regular follow–up, which sometimes may be lifelong. Many of these syndromic patients have a lot of queries regarding their future generation and even their own treatment [3]. All websites do not provide reliable information [4,5]. We hypothesized that both Indian and foreign websites should be similar in all aspects in this era. In this prospective study, we analyzed the websites of Indian endocrine surgeons and whether they were comparable to their foreign counterparts.

## 2 Material and methods

Foreign endocrine surgery websites were identified and assessed based on information provided in the International Association of Endocrine Surgeons' directory [6] and by Google search. The Indian endocrine surgery websites were identified based on the Indian Association of Endocrine Surgeons’ website directory [7].

**Fig. 1:**
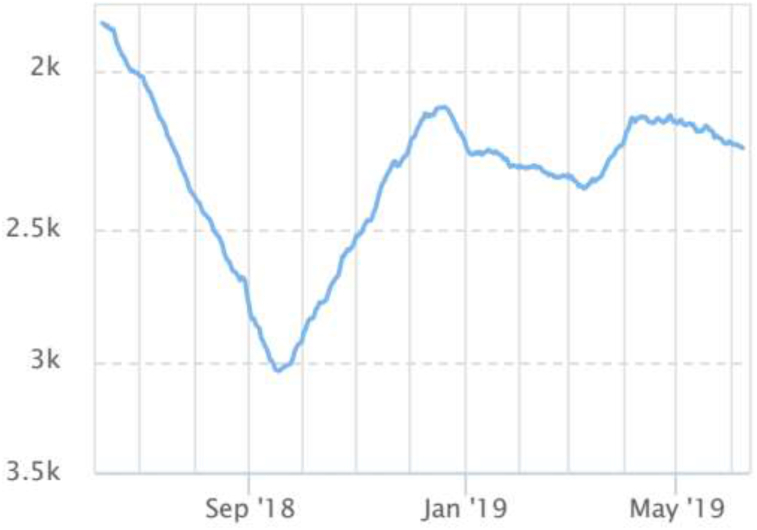
This figures depicts the ranking of websites over 6 months duration relative to other websites.

**Fig. 2:**
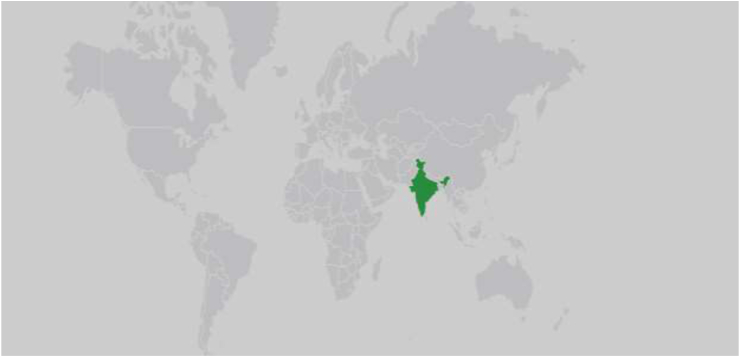
This depicts an Indian endocrine website which is only browsed only by Indians (shaded green). (For interpretation of the references to colour in this figure legend, the reader is referred to the Web version of this article.)

**Fig. 3:**
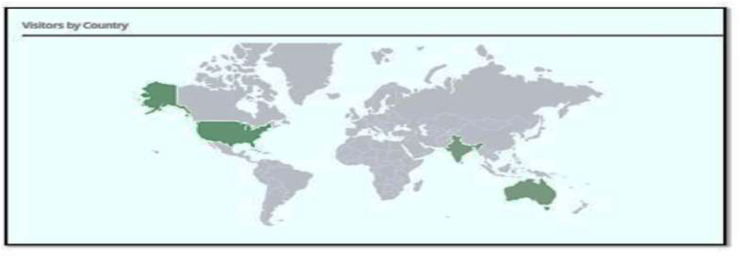
This figure depicts the Endocrine website visitors according to their country and the green shaded area represents the countries where people have browsed this website. (For interpretation of the references to colour in this figure legend, the reader is referred to the Web version of this article.)

A professional website software (www.Alexa.com) [8] was used to assess the ranking number of hits, demographic profile of the website, and other parameters (Fig. 1, Fig. 2, Fig. 3). An endocrine surgeon along with a technical website content developer rated the content of information, presentation of information, and landscape of the website from a scale of 1–5 (1 minimum score and 5 maximum score). The endocrine surgeon also analyzed whether the website had thyroid facts, parathyroid facts, adrenal facts, pancreatic facts, photographs, videos, adequate contact information, postoperative advice, and complications. This work has been reported in line with the STROCSS criteria [9].

Statistical analyses were done using SPSS19.0. Distribution of thyroid facts, parathyroid facts, adrenal facts, pancreatic facts, photographs, videos, adequate contact information, postoperative advice and complications, age groups, and gender in Indian and foreign websites was analyzed by chi square test. Student's *t*-test and Mann Whitney *U* test were used to compare landscape, quality, and presentation of facts between Indian and foreign websites. P values < 0.005 were considered statistically significant.

## 3 Results

A total of 72 individual endocrine surgeon websites, out of which 60 were from abroad and 12 were from India, were analyzed. A majority of the foreign websites were ranked (43/60), whereas 2/12 Indian websites were ranked (P < 0.0001). The age group surfing the website (15–48) was the major user in both groups. Females predominately surfed both foreign and Indian websites. In evaluating the landscape of the website, the foreign websites had a score of 3: 44/60 had a score of 3 (i.e., 86.3%). When comparing the quality of information and the presentation of information, there was no significant statistical difference between foreign and Indian websites (Table. 1).

**Table 1:** Distribution of the demographic and presentation view between indian and foreign websites (N = 72).

Variables	Total (N = 72)	Indian (n = 12)	Foreign (n = 60)	P Value
Age groups (15–48)	48 (66.7)	9 (75)	44 (73.3)	0.999
Gender (M > F)	21 (29.2)	3 (25)	18 (30)	0.999
Landscape				
2	12 (19)	6 (50)	6 (11.8)	0.003
3	49 (77.8)	5 (41.7)	44 (86.3	
4	2 (3.2)	1 (8.3)	1 (2.0)	
Quality				
2	18 (28.6)	1 (8.3)	17 (33.3)	0.142
3	29 (46)	6 (50)	23 (45.1)	
4	16 (25.4)	5 (41.7)	11 (21.6)	
Presentation				
	18 (28.6)	3 (25)	15 (29.4)	0.747
3	35 (55.6)	8 (66.7)	27 (52.9)	
4	10 (15.9)	1 (8.3)	9 (17.6)	

Data are given in frequency (%), ***p* < 0.05 is significant.**

Except for pancreatic facts, which were significantly different (P = 0.006) between Indian and foreign websites, there was no significant difference in thyroid facts, parathyroid facts, adrenal facts, photographs, videos, postop advice, contact information, publications, and complications (Table. 2). However, the complications of surgical procedure were provided only in 39.7% of the websites.

**Table 2:** Distribution of clinical keywords presentation between indian and foreign websites (N = 72).

Variables	Total (N = 72)	Indian (n = 12)	Foreign (n = 60)	P Value
Thyroid facts	58 (80.5)	12 (100)	46 (76.6)	0.062
Parathyroid facts	35 (54.7)	8 (66.7)	27 (51.9)	0.355
Adrenal facts	29 (45.3)	8 (66.7)	21 (40.4)	0.099
Pancreas facts	12 (18.8)	6 (50)	6 (11.5)	**0.006**
Photo graphs	62 (96.9)	11 (91.7)	51 (98.1)	0.342
Videos	34 (53.1)	6 (50)	28 (53.8)	0.810
Postop Advice	30 (46.9)	4 (33.3)	26 (50)	0.297
Contact information	62 (96.9)	11 (91.7)	51 (98.1)	0.342
Publications	62 (86.1)	11 (91.7)	51 (85.0)	0.999
Complications	25 (39.7)	4 (33.3)	21 (41.2)	0.749

Data are given in frequency (%), Chi-square test/Fisher exact test is used.

p < 0.05 is significant.

## 4 Discussion

Our results establish the fact that Indian endocrine surgery websites are comparable to their foreign counterparts. The foreign websites being ranked when compared to Indian websites is because of the fact that these endocrine surgeons are pioneers in the field of endocrine surgery and in India, the superspeciality Mch (formal three year) training started only in 2005. Endocrine surgery is one of the youngest kids in the block in relation to surperspeciality surgical training. The availability of web content developers and web designers is an issue in developing countries, as is the fact that an interactive and well-designed website costs more when compared to a less costly basic website. This might be the reason that the land scape of foreign websites fared well when compared to Indian websites.

The endocrine surgery websites were surfed majority by females, both in Indian websites and foreign websites. This may be due to fact that Thyroid disorders which is the most common endocrine disorders are most common in females. Males did not search predominantly the endocrine surgery website.

In relation to thyroid facts, a previous study by Muthukumuraswary S et al. [4] found that information regarding thyroidectomy was highly variable. They also noted that high ranking and popularity did not correlate with good quality information. We found that the majority of the foreign and Indian endocrine surgery websites had sufficient information regarding thyroidectomy and thyroid disorders.

In a study by McleanT et al. [5], they found that for information regarding minimally invasive parathyroidectomy, 27.3% of the websites had false claims, and these claims were posted to attract surgical referrals. In this study, parathyroid facts were found in 54.7% of the websites. Some endocrine surgeons who subspecialized in parathyroid surgery had considerately more parathyroid facts when compared to endocrine surgeons concentrating on all endocrine organs.

With regard to adrenal surgical facts, the facts were available in 45.3% of the websites. Again, a grey zone where endocrine surgeons have this territory invaded by urologists and surgical oncologists, and the lack of expert training, especially in the retroperitoneoscopic approach during endocrine surgical training, may have had this effect. Endocrine pancreatic tumors are rare, and the territorial occupancy by surgical gastroenterologists is a factor why this information is present only in 18.8% of the websites. Many of these tumors are treated in a tertiary referral center as well.

Photographs of surgery and videos were present in 96.9% and 53% of the websites, respectively. These helped in their clientage. Contact information including email was available in 96.9% of the websites. The main issue was the mention of complications in previously treated patients, which was available in only 39.7%. Most of the time, the complications were not clearly mentioned and the percentage was not available. Patients and their relatives may be misled by these kinds of biases on websites, and the content might not be appropriate for patients with high end information, which may be difficult to understand and comprehend [[10], [11], [12]]. There exists fundamental difference between Indian and foreign websites. These may be due to patient factors, surgeon factors and website factors. The patient factors include the demographic profile, literacy, religious and cultural back ground and economic infrastructure. The surgeon factors include the qualification of the surgeon, the place where trained, academic or private setup and also the nature of the individual. Since many foreign websites are designed by the institute itself and Indian surgeons have to design their website themselves. The qualified personnel available to design website also varies. The financial constraints and also the space available for content storage varies and depends on the affordability.

These websites can be used to provide preoperative information, surgical procedure, complications, and postoperative instructions to be followed [13]. These websites can help in current patient understanding of the disorder and can provide valuable information to the patient and thereby help in much needed focused conversation with the consultant. Interactive websites with different outcomes, videos experiences of previous patients, and app-based patient groups can be of use in the future. This was a pilot study to determine whether Indian websites were comparable to their foreign counterparts and, therefore, did not analyze in depth every issue related to endocrine surgery. Focused issues like robotic thyroidectomy, retroperitoneoscopic adrenalectomy, etc. can be assessed in detail and can educate both the physician and the patient. Still, this study is probably the first of its kind in endocrine surgery.

## 5 Conclusion

Most parameters were comparable in both groups. Complications were present in only a few websites. The advantage of these websites is that patients can gather much needed information, save much of unneeded discussions with physicians, and save on second consultation.

## Author contribution

Authors’ contribution: S.M. and MD contributed to the conception and design of the study. S.M., MD., and DW did the acquisition of data. S.M. and SB. did the analysis and interpretation of data. S.M. drafted the article. All authors revised the article critically for important intellectual content and also the final approval of the version to be submitted.

## Sources of funding for research and/or publication

No Source of Funding

## The category in which the manuscript is being submitted (original article, review, randomized clinical trial)

Original Article.

## Research registration unique identifying number (UIN)

https://www.researchregistry.com/browse-the-registry#home/

## Ethical clearance

Obtained from SGPGI Ethics Committee PGI/BE/304/2019.

## Guarantor

Dr.Mayilvaganan Sabaretnam.

## Declaration of competing interest

Authors have no conflict of interest.
